# Visual Profile of Children who Passed or Failed the UK School Vision Screening Protocol

**DOI:** 10.22599/bioj.121

**Published:** 2019-03-26

**Authors:** Sara McCullough, Kathryn Saunders

**Affiliations:** 1 Ulster University, GB

**Keywords:** vision screening, strabismus, refractive error, amblyopia, sensitivity, specificity

## Abstract

**Background:**

We applied the National Screening Committee vision screening protocol [pass criterion monocular acuity ≤ 0.2 LogMAR in both eyes(BE)] to children four to five years old to investigate the visual profile of children who passed/failed. Previous studies have only evaluated those failing. The aim was to derive false positive and negative values, specificity/sensitivity of the vision screening protocol for detecting significant visual defects (strabismus and/or significant refractive error) and the utility of a ‘plus blur test’ in identifying hyperopia.

**Methods:**

Participants included 294 children (5.2 ± 0.4 yrs). In addition to the vision screening protocol (monocular acuity–3 m crowded Keeler LogMAR letters), acuities were recorded through +2.50D and +4.00D lenses and ocular alignment and cycloplegic refractive error were assessed. Using acuity measures, participants were classed as passing/failing the screening protocol. Each participant was also classed as having a strabismus and/or significant refractive error (hyperopia ≥ +4.00DS; myopia ≤ –0.50DS; astigmatism ≤ –1.50DC; anisometropia ≥ +1.50DS) or no significant visual defects.

**Results:**

Of the 284 children who completed all tests, 27.8% failed to achieve 0.2 LogMAR in BE. The acuity pass/fail criterion had a sensitivity of 70.4% and specificity of 82.2% for detecting strabismus and/or significant refractive error. Of those who failed, 51.9% (n = 41/79) had no strabismus and/or significant refractive error (false positives). Of those who passed, 7.8% (n = 16/205) had visual defects (false negatives). The ‘plus blur tests’ improved sensitivity in detecting significant refractive error (+2.50D & +4.00D 90.7%) but significantly reduced specificity (+2.50D = 65.2%; +4.00D = 60.9%).

**Conclusions:**

School-entry vision screening is reasonably sensitive and specific for detecting strabismus and/or significant refractive error. Most children with visions poorer than 0.2 LogMAR need refractive intervention, and the majority of the remainder are likely false positives for significant visual defects. One in 13 children who pass have either strabismus and/or significant refractive error (7.8%). The inclusion of a ‘plus blur test’ was not a useful addition to the vision screening protocol.

## Introduction

School-entry vision screening aims to detect children with reduced vision. The most common deficits causing reduced vision in children of this age are uncorrected refractive error and strabismus, which can lead to amblyopia if left undetected. Early treatment with glasses or occlusion/penalisation therapy can effectively improve vision; however, if left undetected and untreated, reduced vision can have long-term implications for the child’s education, potential occupation and future vision loss (Chua & Mitchell 2004; Rahi et al. 2002).

The United Kingdom (UK) National Screening Committee (NSC) recommends that all children can be to are screened for reduced vision at school entry (aged four to five years) (UKNSC 2013). The recommendation is for an orthoptic-led service delivered by health professionals, such as a school nurse, orthoptist, health care assistant or specifically trained vision screeners.

Visual acuity is assessed using the Keeler crowded LogMAR test, and children are referred to specialist services for further assessment if they do not achieve 0.2 LogMAR in both eyes. The guidance states that ‘amblyopia is the most likely condition to be detected’ by school vision screening but also suggests that ‘refractive error and strabismus would be detected’ (UKNSC 2013); however, there are few published studies evaluating how well the NSC recommended school vision screening protocol identifies these problems. Neither is it clear from the available literature how many children with significant visual issues pass the recommended screening protocol.

The National Screening Committee have outlined a list of criteria to appraise the viability, effectiveness and appropriateness of screening programmes in the UK (Public Health England 2015). These criteria describe the importance of ‘a plan for managing and monitoring the screening programme and an agreed set of quality assessment standards’; however, there is currently no guidance on what metrics should be used to gauge quality.

Previous studies have described the visual features and treatment needs of children who ‘fail’ vision screening protocols; without exception, they report that the single largest group referred from screening are those with significant uncorrected refractive error without amblyopia or strabismus (Beardsell et al. 1989; Donaldson et al. 2002; Edwards et al. 1989; Fathy & Elton 1993; Jutley et al. 2012; Karas et al. 1999; Kohler & Stigmar 1973; Newman et al. 1996; Williamson et al. 1995). Previous reports also note relatively high proportions of ‘false positive’ referral ranging from 13.3% (O’Colmain et al. 2016) to 43% (Donaldson et al. 2002). However, none of these studies profiled the outcomes derived from the basic NSC-recommended protocol consisting solely of monocular measures of LogMAR crowded acuity at 3 m.

With the recent publication by Public Health England of new child vision screening materials and guidance (Public Health England 2017a), it is timely to investigate the visual profile of children passing and failing the recommended school vision screening protocol. This information can be used to ensure that the diagnostic pathway implemented, and competency frameworks developed to support the pathway, are evidence based and optimise prompt, appropriate treatment. Such an approach is required to meet the NSC screening appraisal criteria that ‘there should be an agreed policy on the further diagnostic investigation with a positive test result’ and that ‘all other options for managing the condition should be considered … to ensure that no more cost effective intervention could be introduced’ (Public Health England 2015).

Given the high number of children reported as failing vision screening with uncorrected refractive error (Bruce et al. 2018), a secondary aim of the present study was to determine whether incorporating an additional assessment of visual acuity with the child looking through a plus (convex) lens may help improve the detection of hyperopia (Bodack et al. 2010; Bosse et al. 1992; Laatikainen & Erkkila 1980; Thomson & Evans 1999; Williams et al. 2005). Viewing through a plus lens should blur the distance vision of those children who are not hyperopic but have little or no impact on children who are hyperopic.

Recent work by the Vision in Preschoolers Hyperopia in Preschoolers (VIP HIP) group has shown that significant levels of uncorrected hyperopia (≥+4.00D in the most hyperopic meridian) are linked to poorer literacy and educational attainment in early school-aged children (Kulp et al. 2016), in addition to the deleterious effect hyperopia has on visual performance at near (Ciner et al. 2016). While school vision screening protocols are designed to detect reduced-distance vision, with the primary aim of identifying potential amblyopes, the inclusion of a plus lens test has been suggested as a useful measure to aid the discovery of children whose visual function, educational attainment and engagement may also be challenged by uncorrected hyperopia. Whilst the use of a plus lens test has been discussed for some time, there is currently a paucity of robust evidence to support the incorporation of such a test into school vision screening protocols and to quantify the most appropriate lens and visual acuity cut-off to employ.

### Aims

The present study investigated the visual profile of children who passed or failed a vision assessment using the UK NSC school vision screening criterion. The primary aims were to determine

1. how many children with the principal significant visual issues (strabismus and/or significant uncorrected refractive error) pass the UK NSC recommended vision screening protocol (false negatives for amblyogenic risk factors),
2. how well the vision screening protocol detects strabismus and/or significant uncorrected refractive error,
3. the sensitivity and specificity of the UK NSC recommended vision screening protocol in identifying reduced vision due to treatable amblyogenic risk factors, and
4. the visual profile of those who fail the vision screening protocol.

Additionally, visual acuity was assessed through positive lenses to determine whether the inclusion of a ‘plus blur test’ alongside the current school vision screening protocol would improve the detection of school-entry children with significant uncorrected hyperopic refractive error. An evaluation of the utility of school vision screening will also be assessed with regards to whether children regularly attend for eye examinations or whether parents/guardians had concerns about their children’s vision or eyes.

## Methods

Fourteen primary schools were approached and agreed to facilitate the study, including seven rural and seven urban. All schools were of mixed gender, were non-selective in academic ability and drew children from a range of socioeconomic backgrounds. Written informed consent was obtained from the parents/guardians of the participants, and verbal assent was given by the participant on the day data collection took place. Data collection took place on school premises during school time. The study was approved by Ulster University Research Ethics Committee (Project number REC/12/0200), and the conduct of the study adhered to the tenets of the Declaration of Helsinki.

### Vision screening assessment

Each child underwent the recommended UK NSC school vision screening protocol of monocular visual acuity assessment using a computerised Keeler crowded LogMAR acuity chart at 3m (UKNSC 2013). The computerised version of the chart was used to standardise background luminance at different testing locations. Other computerised test charts (e.g., Kay pictures) have been shown to be a valid alternative to paper-based test charts in a paediatric population (Shah et al. 2012). The procedure for visual acuity assessment followed the guidelines on the Keeler crowded LogMAR test provided by Public Health England (Public Health England 2018). The screening procedure was used to determine the correct level at which to commence acuity testing. The child then attempted to identify all letters on the initial line, with the examiner proceeding to smaller or larger lines as necessary. At least two letters needed to be correctly identified before decreasing the letter size. All letters were tested on a line where errors occurred; by-letter scoring was applied with each letter equal to 0.025 LogMAR and the final result documented. Acuity was tested to threshold level. Children responded either by naming or matching the letters. The right eye was tested first, followed by the left eye, with the non-tested eye occluded with children’s occlusion glasses. Care was taken to ensure children were not peeking around the occlusion lens. Children who habitually wore spectacles completed all tests unaided. The visual acuity test was carried out by authors SJM or KJS, qualified optometrists with considerable experience of visual acuity testing in children in both research and clinical settings. Acuity measurement was performed prior to all other measures.

Monocular acuity measures were used to identify children who failed the vision screening protocol as set out by the UK National Screening Committee (monocular acuity worse than 0.20 LogMAR in one or both eyes).

### Diagnostic assessment

Each child also underwent the following diagnostic assessments:

- An assessment of ocular alignment at distance and near using the cover/uncover test unaided. The child was directed to fixate on detail within a picture at 3 m and on the Lang Cube at 33 cm.
- Refractive error was assessed using distance autorefraction (Shin-Nippon NVision-K-5001, Shin-Nippon, Tokyo, Japan) at least 30 minutes after the instillation of one drop of 0.5% proxymetacaine hydrochloride followed by one drop of 1% cyclopentolate hydrochloride. The presence of dilated pupils that were nonresponsive to light was used to confirm that cycloplegia had been achieved; where this was not the case, a second drop of 1% cyclopentolate hydrochloride was instilled. No less than five readings were taken from which the representative value as determined by the instrument was used for further analysis. The representative value is widely used as an output value for this instrument and has been shown to be comparable to other methods of averaging refractive error (Tang et al. 2014). Spherical equivalent refractive error (SER) was calculated using sphere + (cylinder/2).

These measures were used to determine which children had significant visual issues. The following were deemed to be significant:

- manifest strabismus
- uncorrected refractive error (either eye most hyperopic meridian ≥ +4.00DS (Ciner et al. 2016; Kulp et al. 2016) either eye myopia spherical equivalent refraction (SER) ≤ –0.50DS; either eye astigmatism ≤ –1.50DS; inter-ocular difference in SER ≥ +1.50DS) (O’Donoghue et al. 2012)

The VIP-HIP Study (Kulp et al. 2016; Ciner et al. 2016) report the greatest deficits of visual function and literacy among children who had ≥4.00D of hyperopia. The Royal College of Ophthalmologists (RCOphth 2012) also suggest that children with hyperopic refractive errors of less than +4.00D do not require glasses. Our cut-off criteria for significant hyperopia have encompassed these publications and guidelines and should be interpreted accordingly; variations in the level of significant refractive error will alter the sensitivity/specificity and false positive/negative results derived.

### Plus blur test

Monocular distance acuities were also measured using the Keeler crowded LogMAR computerised acuity chart at 3m with the child wearing glasses with a plus lens (either +2.50DS or +4.00DS) in front of one eye and an occlusion lens in front of the other eye. All children had their vision assessed with both the +2.50DS and the +4.00DS lens for both eyes. The time taken to deliver the ‘plus blur test’ was recorded using a stopwatch.

### Parent report of children’s visual health and vision screening results

When giving written consent for their child to participate in the study, parents/guardians were also asked to complete a short questionnaire. This included information on previous eye examinations, family history of eye/vision problems and whether the parent/guardian had any concerns about their child’s vision. (Supplementary material 1)

After the study was completed and all children had completed their first year of formal education, parents/guardians were re-contacted to ask them to report the result of their child’s school vision screening test carried out in school by the school nurse. Responses were provided by completing a reply letter and returning it to the study coordinator in a Free-post envelope. (Supplementary material 2)

### Statistical analysis

All statistical analyses were carried out using Stata Intercooled 13.0 (StataCorp, Texas, USA). Sensitivity and specificity values were calculated for the ability of the UK NSC school vision screening protocol to detect strabismus and/or significant refractive error. The visual profile of the children who passed or failed the school vision screening protocol was also determined. Receiver Operating Characteristic (ROC) curves were examined to ascertain the best cut-off point (taken as the point closest to the top left-hand corner of the ROC curve) of visual acuity with the ‘+2.50D and +4.00D blur tests’ to detect significant hyperopia. These visual acuity cut-offs were used in addition to the current school vision screening to determine whether the combination of the tests improved the detection of those children with significant hyperopia, as well as strabismus and/or other significant refractive errors. Chi-squared analysis was used to assess agreement between the pass/fail classifications assigned to participants by the current study group compared with that of the school nurse’s assessment. Mann-Whitney tests were used to assess differences in age between children who passed or failed the vision screening protocol.

## Results

A total of 294 typically developing children in their first year of formal education in Northern Ireland (Kindergarten equivalent) were recruited, with a participation rate of 36%. The median age of participants was 5.23 years (range 4.0 to 5.9 years), with a gender mix of 45% male and 55% female. The majority of participants were white (n = 278, 95%) consistent with the demographics of the Northern Irish population (NISRA 2011).

All participants (n = 294, 100%) were compliant with the measurement of monocular distance visual acuities. Complete acuity and diagnostic data were available for 284 participants (96.6%) (Table 1). Eighteen participants were unable to complete monocular distance visual acuities with the ‘+2.50DS and +4.00DS blur tests’ (data available for n = 276, 93.9% of those recruited).

**Table 1:** Visual assessment results. Those classified with astigmatism are those children not already classified with myopia or hyperopia. Those classified with strabismus are also re-classified in the Significant Refractive Error section.

Visual Acuity and Diagnostic Assessment Results	N (%)	Passed Screening Protocol N (%)	Failed Screening Protocol N (%)

**Passed UK NSC screening protocol** Distance VA ≤ 0.2 LogMAR either eye	**205 (72.2)**		
**Failed UK NSC screening protocol** Distance VA > 0.2 LogMAR either eye	**79 (27.8)**		
Distance VA ≥ 0.5 LogMAR either eye	15 (5.3)		
Difference in VA of ≥0.5 LogMAR	6 (2.1)		
**Strabismus (Total)**	**9 (3.2)**	**2 (22.2)**	**7 (77.7)**
Esotropia	5 (1.8)	0 (0)	5 (100)
Exotropia	3 (1.1)	2 (66.7)	1 (33.3)
Hypertropia	1 (0.3)	0 (0)	1 (100)
**No Strabismus**	**271 (96.8)**		
**Significant Refractive Error (Total)**	**52 (18.3)**	**15 (28.8)**	**37 (71.2)**
Myopia SER ≤ –0.50D	1 (0.4)	0 (0)	1 (100)
Hyperopia MAM ≥ +4.00D	41 (14.4)	13 (31.7)	28 (68.3)
Astigmatism Cyl ≤ –1.50D	10 (3.5)	2 (20)	8 (80)
Anisometropia Diff in SER ≥ +1.50D	9 (3.2)	1 (11.1)	8 (88.9)
**No Significant Refractive Error**	**232 (81.7)**		

Table 2 shows the sensitivity and specificity of the UK NSC vision screening protocol for detecting strabismus and/or significant refractive error.

**Table 2:** Sensitivity and specificity of the UK NSC vision screening protocol (vision assessment using the Keeler crowded LogMAR acuity chart) at detecting children with strabismus and/or significant refractive error. EE = either eye.

	Strabismus and/or significant refractive error Present (n)	Strabismus and/or significant refractive error Absent (n)

**Fail**	38	41
**(>0.2 LogMAR EE)**		
**Pass**	16	189
**(≤0.2 LogMAR EE)**		
	**Sensitivity = 70.4%**	**Specificity = 82.2%**

The majority of children who passed the vision screening protocol (n = 189/205, 92.2%) were found in the diagnostic assessment to have no potentially amblyogenic risk factors. Sixteen children had strabismus and/or significant refractive error and were found to pass the screening protocol (n = 16/205, 7.8%); the majority of these had significant uncorrected refractive errors (n = 15/16), predominantly hyperopia (n = 13/16) (most ametropic eye, median +4.50DS, range +4.00DS to +8.00D). One child had significant anisometropia with significant astigmatism and a visual acuity difference between their eyes of 0.10 LogMAR (RE 0.05 LogMAR, LE 0.15 LogMAR). Two children passing the screening protocol had manifest strabismus, one with alternating exotropia and one with a left distance exotropia.

### What is the visual profile of those who fail the vision screening protocol?

Of the 79 children whose visual acuity was worse than 0.2 LogMAR in either eye, strabismus and/or significant refractive error were present in 38 children (48.1%) and were likely to explain the reduced acuity measure. The majority of these children had significant uncorrected refractive error only (n = 31/38, 81.6%). Seven children (8.9%) whose acuity was poorer than 0.2 LogMAR had strabismus, and six of these children had a significant co-existing refractive error. The visual profile of 51.9% of participants (n = 41/79) who had a visual acuity of worse than 0.2 LogMAR did not indicate any need for clinical intervention and demonstrated no significant visual issues to explain the poor acuity measure. The median sphere and cylinder for both eyes was 2.25D (range 0.25 to 3.75D) and –0.50DC (range 0.00 to –1.25DC), respectively. The median visual acuity of these children was 0.25 LogMAR for both eyes, which equates to approximately two letters poorer than the criteria required to pass the acuity test. The median age for these children was 5.1 years (IQR 4.2 to 5.8 years) compared to 5.3 years (IQR 4.3 to 5.8 years) for those children who passed the screening protocol and did not indicate a need for clinical intervention; this difference was statistically significant (Mann-Whitney analysis, z = 2.27, p = 0.02). Overall, children who failed the school vision screening protocol were statistically significantly younger (median = 5.1 years, IQR 4.8 to 5.4 years) than those who passed (median = 5.3 years, IQR 5.0 to 5.8 years) (Mann-Whitney analysis z = 2.18, p = 0.03).

### Parent questionnaire

Parent questionnaire data were available from 279 participants. Of these participants, 89 parents reported taking their child for an eye examination within the last year (31.9%).

**Figure 1:**
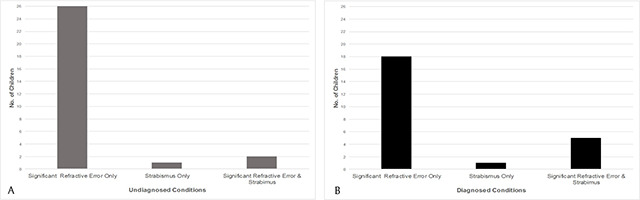
Frequency of undiagnosed visual defects **(A)**. Frequency of diagnosed visual defects **(B)**.

A number of significant visual defects were previously undiagnosed (n = 29/53, 54.7%), with the majority of these being significant uncorrected refractive error alone (n = 26/29, 89.7%) (Figure 1A) (data were missing from one parent questionnaire where the child had a significant refractive error; therefore, we cannot comment whether it was diagnosed or not). Most of the parents/guardians of these children had no concerns about their child’s vision/eyes (n = 26/29, 89.7%), and most (n = 28/29, 96.6%) had not taken them for an eye examination within the last year. Details of the visual defects (significant refractive error and/or strabismus) that were diagnosed and undiagnosed before the child attended the vision study are presented in Figure 1A and 1B. The diagnosed visual defects in Figure 1B are calculated from the details provided in the parent questionnaire on whether the child currently wore spectacles or if the child had strabismus.

### School vision screening result carried out by school nurse/vision screener

One hundred and four parents/guardians (37%) responded to the request for information about their child’s performance in the school vision screening carried out by the school nurse/vision screener during the first year of formal education. This may have occurred prior to or after their participation in the present study. Three (n = 3/104, 2.9%) parents/guardians reported their child ‘did not take part’, and five (n = 5/104, 4.8%) reported their child was ‘not offered to take part’ in school vision screening. Twenty (n = 20/104, 19.2%) parents/guardians reported they were ‘unsure of the result’ of their child’s school vision screening. A comparison of the child’s school vision screening result according to parental report to the results of the vision assessment carried out within the present study is outlined in Table 3 for the remaining 77 participants. There was significant agreement between the outcomes of the two vision assessments for 90.9% of the children (Chi-squared = 44.2, p < 0.0001).

**Table 3:** Comparison of the outcomes from school vision screening by the school nurse (according to parental report) and the results of the visual acuity test in the present study, n = 77.

		School vision screening result according to parental report	

		Pass	Fail

**Result of acuity test in present study**	**Pass**	58	1
	**Fail**	5	12

### Detection of hyperopia through ‘plus blur tests’

**Figure 2:**
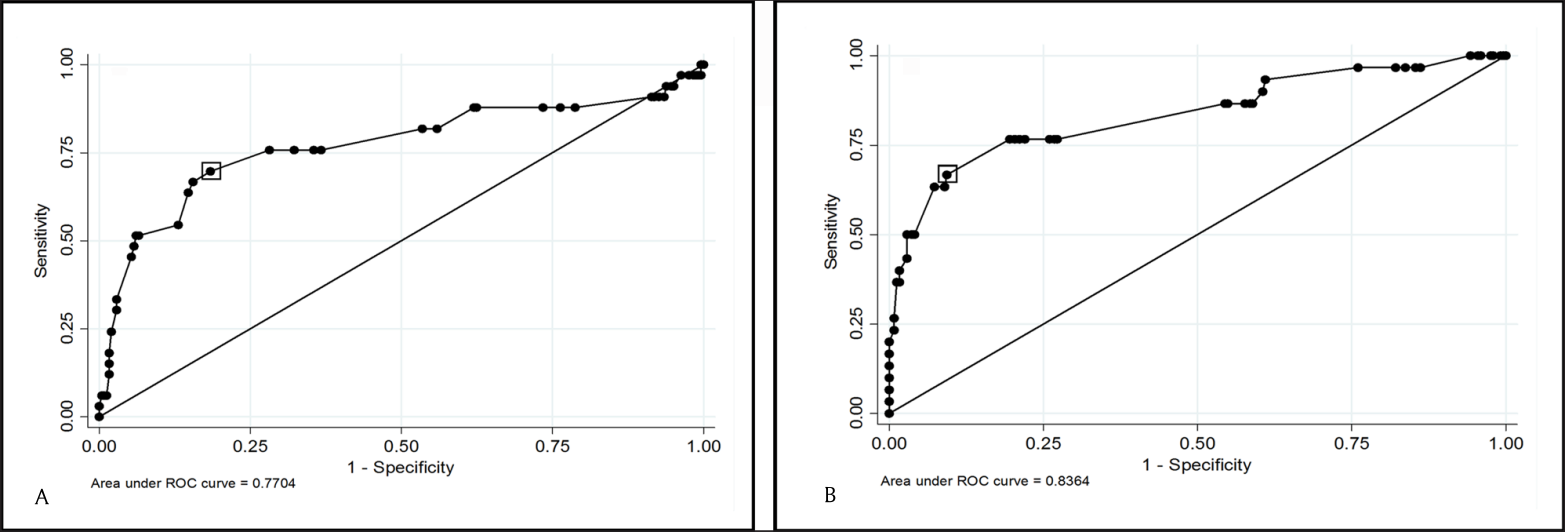
ROC Curves: Use of ‘+2.50D blur test’ to detect significant hyperopia (≥+4.00D). The boxes highlight the best visual acuity cut-off points that relate to the right (sensitivity 69.7%, specificity 81.63%) **(A)** and left eyes (sensitivity 70.00%, 83.87%) **(B)**.

**Figure 3:**
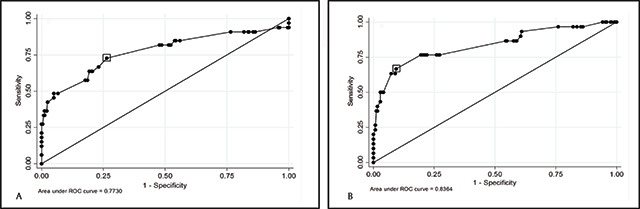
ROC Curves: Use of ‘+4.00D blur test’ to detect significant hyperopia (≥+4.00D). The boxes highlight the best visual acuity cut-off points that relate to the right (sensitivity 72.73%, specificity 73.66%) **(A)** and left eyes (sensitivity 66.67%, 90.65%) **(B)**.

Receiver Operator Characteristic (ROC) curves were generated to explore the best visual acuity cut-off point for the detection of significant hyperopia using +2.50D and +4.00D lenses for the right and left eyes (Figures 2A, 2B, 3A and 3B, respectively).

For the ‘+2.50D blur test’, the best visual acuity cut-off point for detecting hyperopia using ROC curve analysis was ≥0.675 LogMAR for the right eye and ≥0.60 LogMAR for the left eye. An average ≥0.638 LogMAR was taken as the best visual acuity cut-off for the two eyes. For the ‘+4.00D blur test’, the best visual acuity cut-off point for detecting hyperopia was ≥1.067 LogMAR for the right eye and ≥0.975 for the left eye. An average ≥1.021 LogMAR was taken as the best visual acuity cut-off for the two eyes. These values were used in addition to the current vision screening protocol (visual acuity poorer than 0.2 LogMAR in either eye) to assess whether the addition of a ‘plus blur test’ was valuable to detect children with significant hyperopia at the time of school vision screening. The failure criteria included

1. Visual acuity >0.2 LogMAR in either eye or visual acuity with ‘+2.50D blur test’ of ≤0.638 LogMAR in either eye.
2. Visual acuity >0.2 LogMAR in either eye or visual acuity through ‘+4.00D blur test’ of ≤1.021 LogMAR in either eye.

Table 4 shows the sensitivity and specificity using failure criteria 1.) and 2.) and the current vision screening protocol for comparison.

**Table 4:** Sensitivity and specificity of failure criteria 1.) and 2.) and the current screening protocol at detecting children with strabismus and/or significant refractive error.

		Strabismus and/or significant refractive error

**Current Protocol**	**Sensitivity (%)**	70.4
	**Specificity (%)**	82.2
**Criteria 1.)**	**Sensitivity (%)**	90.7
	**Specificity (%)**	65.2
**Criteria 2.)**	**Sensitivity (%)**	90.7
	**Specificity (%)**	60.9

Using the additional ‘+2.50D blur test’ or the ‘+4.00D blur test’ identified a further 11 participants with strabismus and/or significant refractive error who were not detected by the UK NSC school vision screening protocol. However, 39 additional false positive results were generated with the ‘+2.50D blur test’ and an additional 49 with the ‘+4.00D blur test’.

It took, on average, an additional 93 seconds (range 40–199 secs) to measure visual acuities of both eyes with the ‘+2.50D blur test’ and an additional 80 seconds (37–222 secs) with the ‘+4.00D blur test’.

## Discussion

While previous studies have reported the visual profiles of children failing the UK NSC recommended school vision screening protocol (e.g., O’Colmain et al. 2016; Bruce et al. 2018), the present study also reports, for the first time, the profile of those who pass the screening protocol. These data allow estimation of the vision screening protocol’s false negative value and sensitivity and specificity in identifying treatable amblyogenic risk factors, such as strabismus and/or significant refractive error. The majority of children who passed the UK NSC vision screening protocol, achieving 0.2 LogMAR or better in both eyes, in the present study (92.2%) were not found, on further examination, to have either strabismus and/or significant refractive error. However, 7.8% who passed the acuity test did have one of these significant visual defects. Although these children are unlikely to need amblyopia treatment as their level of vision falls within normal limits for their age, identification and correction of these visual issues would be of benefit to the child for future visual and educational development. These figures suggest the UK NSC vision screening protocol has moderately good sensitivity (70.4%) and specificity (82.2%) for detecting strabismus and/or significant refractive error. To the authors’ knowledge, these figures have not previously been available for the current UK NSC school vision screening protocol.

Children who were deemed false negatives, having passed the vision screening protocol, had significant refractive errors ranging from +4.00D to +8.00D (n = 15/16), significant astigmatism and anisometropia (n = 1/16), and strabismus (n = 2/16). The false negative figure derived (7.8%) is determined by the criteria chosen to indicate significant refractive error. The criteria chosen in the present study are conservative, in line with prescribing guidance from the Royal College of Ophthalmologists. If less conservative criteria are applied (e.g., Bruce et al. 2018), false negative rates will increase (and correspondingly false positives will decrease).

While it is not possible to directly compare sensitivity and specificity across vision screening protocols with different criteria for inclusion of significant refractive error, our figures compare favourably with outcomes reported by the Vision in Preschoolers (VIP) Study using crowded LogMAR acuity charts to detect refractive error (≥5.00D hyperopia, ≥2.50 astigmatism, myopia ≤ –6.00D, severe anisometropia = interocular difference >2D hyperopia, >3D astigmatism, or >6D myopia) and strabismus among children aged 3–5 years in the United States (US). The crowded LEA symbols chart administered by school nurses in the US showed a 49% sensitivity when specificity was set to 90% (Vision in Preschoolers Study Group 2005), and the crowded HOTV acuity chart showed 54% sensitivity and 89% specificity when administered by eye care professionals (Schmidt et al. 2004). The lower sensitivity found in the VIP study compared to the current study is likely due to the higher categories set for significant refractive error.

Amongst participants in the present study, almost 55% (n = 29/53) of those who were found to have a visual defect at age 4–5 years were undiagnosed (10.2% of the study group), and 55% of these (n = 16/29) would have been identified by the standard NSC school vision screening protocol. Recent coverage in the optometric media (Association of Optometrists 2017) has reported that one in five school-aged children have an undiagnosed vision problem within the UK. Our results suggest this figure is closer to one in ten among children of school-entry age. However, we acknowledge that, with increasing age, the number of children with myopia will increase and potentially inflate this figure (O’Donoghue et al. 2010; Breslin et al. 2013; McCullough et al. 2016).

Within our study group, just over 30% of children had undergone an eye examination within the last year. This figure is higher than that reported by Guggenheim and Farbrother (2005), who reported that only 7% of children under the age of five years living in the North of England had visited their optometrist for an eye examination within the last year. However, the fact that 70% of the children within our study had not had an eye examination within the last year highlights the importance of continuing universal coverage of vision screening at school entry. It is also important to make parents/guardians of children who pass school vision screening aware that screening, by its nature, cannot detect all vision problems (7.8% of those who passed had significant visual issues). Following the recent consultation on school vision screening, Public Health England recommend that post-screening information letters given to parents/guardians of children who pass should identify the limitations of school entry vision screening and highlight the availability and importance of ongoing eye care through childhood (Public Health England 2017b).

The percentage of children failing to achieve acuities of 0.2 LogMAR or better in both eyes was 27.8% within the present study. Published failure rates for school vision screening protocols in the UK (2015/16) range from 4–24%, with an average of 12% (Carlton et al. 2017); the failure rate of the present study is at the high end of this range. The age profile of children participating in screening programmes is likely to influence the failure rate. Those children who failed the vision screening protocol in the absence of potentially amblyogenic risk factors were significantly younger than those children who passed. The median vision measurement achieved for both eyes for these children was 0.25 LogMAR, two letters worse than the pass criterion. This highlights that younger children are more likely to fail vision screening even when age appropriate vision tests are used with matching cards available.

All children within the present study were able to complete the crowded LogMAR letter test, which is considered to be the most sensitive for screening for amblyopia (Simmers et al. 1997). Acuity measurement undertaken by eye care clinicians, rather than school nurses, may have inflated our failure rate. The Vision in Preschoolers Study (Vision in Preschoolers Group 2009) report an increased failure rate when experienced clinicians carry out the vision assessment compared to lay screeners. The higher failure rate could have inflated the number of false positive results presented; however, in the main, our pass/fail classification of participants is in agreement with that conducted by the school nurse screener.

Significant refractive error alone was found to be the predominant cause for a child to have reduced vision under the current screening protocol (39.2%, n = 31/79). Seven children who failed (8.9%) were found to have strabismus, and all but one of these children had significant uncorrected refractive error. More than half of the children who failed the vision assessment within the current study had no significant visual issues to explain reduced visual acuities (51.9%). Donaldson et al. (2002) reported on a secondary vision screening service that triaged children who failed school vision screening using a joint optometric/orthoptic assessment. They report similar figures; 43% of the children failing initial screening were found to be ‘visually normal’ and were discharged without treatment, and 41% of children required refractive correction as the only treatment. Only 16% of children required onward referral to the Hospital Eye Service. Donaldson et al.’s findings and those of the present study are echoed by other published data [5–13]. A recent paper by Bruce et al. (2018) report a false positive rate of 7% for their study population of children based in Bradford, UK, which is low in contrast to the results of the present study. However, almost 40% (n = 953) of the children who failed the vision assessment did not attend for their follow-up visit to determine whether a visual defect was present or not. Bruce et al.’s threshold for significant hyperopia is also considerably lower (≥+2.00D SER) than that used within the present study. A higher level of hyperopia (≥+4.00D sphere) was chosen in the present study to reflect prescribing guidance from the Royal College of Ophthalmologists and to mediate against inflation of false negatives; in turn, this may have increased the number of false positives. Lowering the criteria for significant hyperopia to ≥+2.00D SER would result in a false positive result of 29.1% (n = 23/79), which still remains significantly greater than that reported by Bruce et al. In the context of quality improvement frameworks for healthcare (e.g., The Health Foundation 2013), such data should be utilised to develop service delivery models that prioritise the assessment and treatment of refractive error for children who fail vision screening diagnostic pathways such that children’s visual needs are identified and met in a timely and efficient fashion.

The addition of the ‘+2.50D and +4.00D blur tests’ improved the sensitivity of the screening protocol for the detection of significant refractive error and strabismus; however, the specificity was significantly reduced with both ‘plus blur tests’. A substantial number of false positives would be generated if added to the current vision screening protocol recommendations. Additionally, administration of each ‘plus blur test’ added an average of 80–90 seconds to the assessment duration for each child. The extra time taken to administer the test, together with the poor specificity values achieved, suggest that the ‘plus blur tests’ may not be a useful addition to the recommended school entry vision screening protocol.

The study presents data from a large number of typically developing children who underwent the recommended UK NSC school vision screening protocol. We have assessed for the first time the presence of visual defects among those children who pass the school vision screening protocol and have identified the percentage of false negatives generated. The study conducted a diagnostic assessment on all the participants to establish refractive error under cycloplegia and oculomotor status. However, we did not assess the integrity of the fundus; therefore, some children may have been incorrectly identified as a false positive where a fundal abnormality may have been the cause of reduced visual acuity. Given the extremely rare nature of such deficits, we expect this omission to have had minimal impact on our findings (Pollack & Brodie 1998).

The results of our judgement on whether the child’s visual performance should pass or fail the vision screening test was comparable to that carried out by the school vision screening service in our area, with the majority of the results in agreement (90%). However, we had access to limited data on the screening outcome as only 37% of parents/guardians responded to our request to share the outcome of their child’s school vision screening.

## Conclusions

The UK NSC school vision screening protocol has moderately good sensitivity and specificity for the detection of strabismus and/or significant refractive error, and inclusion of plus blur lenses doesn’t add significant value. More than half of the children who failed the vision screening protocol had no apparent visual defect and were regarded as false positives. Younger children were more likely to contribute to this figure. Significant refractive error was the primary cause of reduced visual acuity.

One in 13 children who passed the school vision screening protocol had a significant visual defect (false negative 7.8%). In line with Public Health England’s updated vision screening recommendations, our data support the need for clear advice for parents/guardians regarding the limitations of vision screening and, in the UK context, making them aware of the availability of the free NHS eye examination, particularly where concerns about their child’s vision or eyes exist.

## Additional File

The additional file for this article can be found as follows:

10.22599/bioj.121.s1 Supplementary material 1. Pre – appointment information.

10.22599/bioj.121.s2 Supplementary material 2. School nurse screening result.
