# Supplementary material for: Visual Profile of Children who Passed or Failed the UK School Vision Screening Protocol
Source: Br Ir Orthopt J. 2019 Mar 26;15(1):36–46. doi: 10.22599/bioj.121 (PMC7510406; doi:10.22599/bioj.121)
Supplement: Supplementary material 1. — Pre – appointment information. [file bioj-15-1-121-s1.pdf]

Supplementary material 1.

Pre – appointment information

1) **Has your child had an eye test within the past year?**
   Yes ☐ No ☐

2) **Does your child wear glasses?**
   Yes ☐ No ☐ Supposed to ☐ Previously worn ☐

3) **Does your child wear a patch?**
   Yes ☐ No ☐

4) **Does your child have a squint?**
   Yes ☐ No ☐

5) **Do you have any concerns about your child's vision?**
   Yes ☐ No ☐

   **(if yes please expand in the box below)**

5) **Is there any family history of eye sight problems?**
   Yes ☐ No ☐

   **(if yes please expand in the box below)**

Thank you for taking the time to answer these questions. If you have any further questions or comments you feel would be helpful please complete them in the box below.
