# Supplementary material for: Visual Profile of Children who Passed or Failed the UK School Vision Screening Protocol
Source: Br Ir Orthopt J. 2019 Mar 26;15(1):36–46. doi: 10.22599/bioj.121 (PMC7510406; doi:10.22599/bioj.121)
Supplement: Supplementary material 2. — School nurse screening result. [file bioj-15-1-121-s2.pdf]

NAME

DOB: XXXX

School: XXXX

Please tick (✓) the box that applies.

Your child:

- **Passed the school vision screening test with the School Nurse** ☐

  (met the standard of vision, no further review by the school nurse)

- **Did not pass the school vision screening test with the School Nurse** ☐

  (required further vision testing with an optometrist/optician or orthoptist, a referral form would have been included in your letter from the School Nurse)

- **Did not take part in school vision screening with the School Nurse** ☐

- **Was not offered school vision screening with the School Nurse** ☐

OR

- **I am unsure of the result of my child's vision screening test with the School Nurse** ☐
